# Shear Bond Strength of Metal and Ceramic Brackets Depending on Etching Protocol in Direct Bonding Technique

**DOI:** 10.3390/ma16206697

**Published:** 2023-10-15

**Authors:** Agnieszka Nawrocka, Joanna Nowak, Salvatore Sauro, Louis Hardan, Rim Bourgi, Monika Lukomska-Szymanska

**Affiliations:** 1 Department of General Dentistry, Medical University of Lodz, 251 Pomorska St., 92-213 Lodz, Poland; agnieszka.nawrocka@stud.umed.lodz.pl; 2 University Laboratory of Materials Research, Medical University of Lodz, Pomorska 251, 92-213 Lodz, Poland; joanna.nowak.1@umed.lodz.pl; 3 Dental Biomaterials, Preventive and Minimally Invasive Dentistry, Departamento de Odontología, Facultad de Ciencias de la Salud, Universidad CEU-Cardenal Herrera C/Del Pozo ss/n, Alfara del Patriarca, 46115 Valencia, Spain; salvatore.sauro@uchceu.es; 4 Department of Therapeutic Dentistry, I.M. Sechenov First Moscow State Medical University, Moscow 119146, Russia; 5 Department of Restorative Dentistry, School of Dentistry, Saint-Joseph University, Beirut 1107 2180, Lebanon; louis.hardan@usj.edu.lb (L.H.); rim.bourgi@net.usj.edu.lb (R.B.); 6 Department of Biomaterials and Bioengineering, INSERM UMR_S 1121, University of Strasbourg, 67000 Strasbourg, France

**Keywords:** adhesion, adhesive remnant index, ARI, ceramic brackets, etch-and-rinse technique, metal brackets, orthodontic brackets, SBS, self-etch technique, shear bond strength

## Abstract

Successful orthodontic therapy, apart from a proper treatment plan, depends on optimal bracket–enamel adhesion. Among numerous factors affecting adhesion, the type of bracket and preparation of the tooth’s surface are crucial. The aim of this study was to compare the shear bond strength (SBS) of metal and ceramic brackets to the enamel’s surface using direct bonding. Forty extracted human premolars were divided into four groups according to the etching method (etch-and-rinse and self-etch) and bracket type. The SBS and adhesive remnant index (ARI) were determined. The ceramic brackets achieved the highest SBS values both in the self-etch (SE) and etch-and-rinse (ER) protocols. Higher SBS values for ceramic and metallic brackets were found in the ER protocol. In all tested groups, the achieved SBS value was satisfactory to withstand orthodontic and occlusal forces. There was no significant difference in the ARI score between study groups (*p* = 0.71). The fracture occurred between the bracket base and adhesive material in both types of brackets, which decreased the risk of enamel damage during debonding.

## 1. Introduction

Orthodontic brackets are a fundamental element of fixed appliance treatment. Being attached to vestibular or lingual tooth surfaces, they deliver the forces induced by archwires, springs or elastics, causing the movement of teeth [1]. Modern orthodontic brackets must meet numerous demands, such as corrosion resistance, reduced plaque accumulation, acceptable aesthetics and low friction in the bracket–wire interaction. Particularly important is their adhesion to the enamel’s surface. The adhesion between the enamel and bracket should be strong enough to resist orthodontic forces, but also safe for the enamel during debonding. Sufficient bracket adhesion reduces the incidence of undesirable debonding, decreases the risk of complications and does not prolong treatment time [1,2,3].

Along with an individual’s treatment plan, bracket placement is the most important procedure affecting the results of orthodontic therapy. Before the bonding procedure, the clinician should take into consideration numerous aspects that are responsible for successful bonding [4,5]. The first factor is the bracket design (including the material and bracket base characteristics) [1,6]. 

Metal brackets made of stainless steel are most frequently used in orthodontic treatment [7,8]. They have some advantages, namely, a low cost of manufacturing, sufficient strength to withstand orthodontic forces and proper corrosion resistance. However, these brackets exhibit poor aesthetics, a rough superficial structure responsible for easier plaque accumulation and low biocompatibility due to the nickel content [1,7,8]. 

Ceramic brackets were invented and developed to meet the need for the enhanced aesthetics of orthodontic appliances [1]. Predominantly, these brackets are made of aluminium oxide (alumina) and are available in two forms—monocrystalline (sapphire) and polycrystalline. Both types of brackets are derived from high-purity corundum ceramic including >99.5% (vol%) Al_2_O_3_. The other components, described as modifiers, are added in trace amounts to the alumina powder to facilitate the manufacturing process. For instance, >0.5% (vol%) MgO reduces the amount of oversized crystals. The addition of MnO and TiO_2_ decreases the sintering temperature [1]. Ceramic brackets are biocompatible and corrosion-resistant. However, because of their increased hardness (greater than that of enamel), they may cause tooth attrition. On the other hand, ceramic brackets are brittle and can be easily fractured during ligation, especially with a heavy orthodontic wire. Altogether, an improper debonding procedure might not only damage brackets, but also pose a risk to the enamel’s surface [8,9,10,11]. 

Depending on the bracket material, various methods are used to achieve their optimal bond with a tooth’s surface [1]. A metal bracket base does not chemically react with adhesive resin, and as a result, the attachment (bracket base–adhesive) relies on micromechanical retention [1,6,9,10,11,12,13,14,15]. Thus, the micromechanical retention depends on the design of the bracket base. Currently, the most common one is a mesh type that can be characterized by perimeters such as the mesh gauge, number of mesh layers and the mesh wire diameters [1,9]. 

On the other hand, to achieve the proper retention of ceramic brackets, undercuts and grooves are created with lasers in the bracket base to provide mechanical interlocking with the adhesive. The bonding process for both types of ceramic brackets is similar and is based on the same mechanism as is used in metal brackets. Surface irregularities extend the contact area between adhesive and ceramic brackets (without the need to increase the overall bracket size) and enhance bonding. Internal edges in the grooves should be 90° and have small intercuts; otherwise, the bracket could slide along the groove [1]. Another solution to improving the adhesion is micromechanical retention, which may coexist with mechanical grooves or occur separately on the bracket’s base. In ceramic brackets, this is a layer or irregular glass particles emerging from the bracket during sinterization. In monocrystalline brackets, this is just an additional thin layer of crystals added to the base during the manufacturing process [1]. According to the literature, mechanical and micromechanical retention is currently recommended [1,9,16]. The reason behind this is that the chemical retention (the silanization of the bracket base) used in clinical trials has caused serious enamel damage during debonding and as a consequence, should be discarded [1,16]. 

Apart from the material and bracket base design, the bracket base area is an important factor in achieving an optimal bond strength. A larger surface is related not only to better adhesion but also to an enhanced control of tooth rotation and in–out (bucco-lingual movement) expression. Depending on manufacturer and bracket material, the base area ranges between 8.9 and 28.5 mm^2^ [1,4,6]. The main concern of having an increased bracket base area is worsened aesthetics and possible occlusal interferences. As a consequence, larger brackets cannot be applied on teeth with short or/and narrow clinical crowns [4]. 

The second factor responsible for successful bonding is the preparation of the enamel’s surface. In order to create micromechanical retention on the enamel’s surface, before the application of a resin-based adhesive, the etching procedure is performed. The etch-and-rinse technique (ER) is the gold standard in dental bonding. After 30 s of exposure to 35–37% orthophosphoric acid, the enamel gains a favourable structure. Because of the selective dissolution of enamel prism cores or their peripheries, adhesive that is subsequently applied can penetrate into surface irregularities [10,17,18,19,20]. On the other hand, the self-etching (SE) technique in orthodontics significantly reduced the chair-side time and risk of moisture contamination of the enamel’s surface during bracket placements [18]. The acidic monomers in the SE adhesive enabled the simultaneous etching and priming stage. The etching potential of the SE technique is lower in comparison to that of ER due to its limited ability to dissolve enamel prisms [21,22]. As a result, the shear bond strength (SBS) values are lower, but still sufficient for effective orthodontic treatment [10,17,18]. 

Thirdly, the direct or indirect bonding technique can be applied. Conventionally, a bracket base with adhesive is placed on a prepared enamel surface (using the SE or ER protocol). In this method, each bracket is positioned directly on the tooth. In contrast, in the indirect bonding method, the appliance position is planned, fixed on a plaster model and transferred with a custom-made transparent tray into the oral cavity [23]. 

Among numerous factors affecting the adhesion between the enamel and bracket, three (the choice of the bracket type, the etching protocol and bonding technique) can be controlled by orthodontists. The balance between the undestroyed bracket–enamel bond (subjected to continuous orthodontic forces) and post-treatment enamel-safe debonding is of crucial importance. Thus, the aim of the present paper was to evaluate the SBS of metal and ceramic brackets bonded directly to the enamel’s surface depending on the etching protocol (ER or SE). The null hypothesis was that there was no significant difference in the SBS value between metal and ceramic brackets bonded either with the SE or ER protocols. 

## 2. Materials and Methods

### 2.1. Study Groups

Firstly, forty human premolars extracted for orthodontic purposes were embedded in cylindrical blocks made of self-cured adhesive resin (Duracryl Plus, SpofaDental, Jicin, Czech Republic). The buccal surfaces of the teeth projected above the acrylic to be accessible to the bonding procedure. The specimens were cleaned with non-fluoride paste (Clean Polish, KerrHawe, Bioggio, Switzerland), rinsed and dried thoroughly with a dental air spray syringe. Specimens were divided into four study groups (*n* = 10) according to etching technique and bracket type, as shown in Table 1.

Next, DB procedure was performed using materials presented in Table 2. The standardized amount of adhesive (2 mm of material measured with endodontic ruler) was spread on the bracket base to cover the whole surface [4].

**Figure 1:**
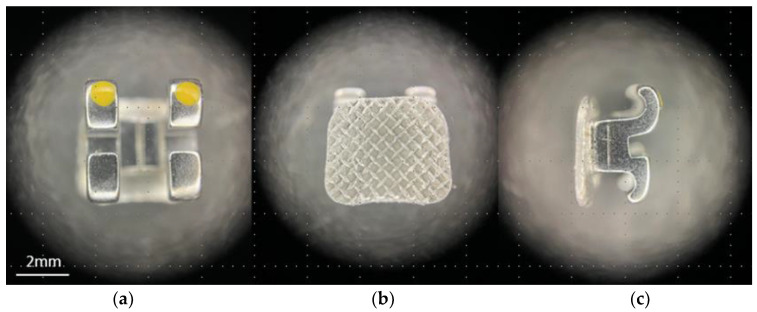
Metal bracket with mesh type base: (**a**) frontal view; (**b**) bracket base; and (**c**) lateral view.

**Figure 2:**
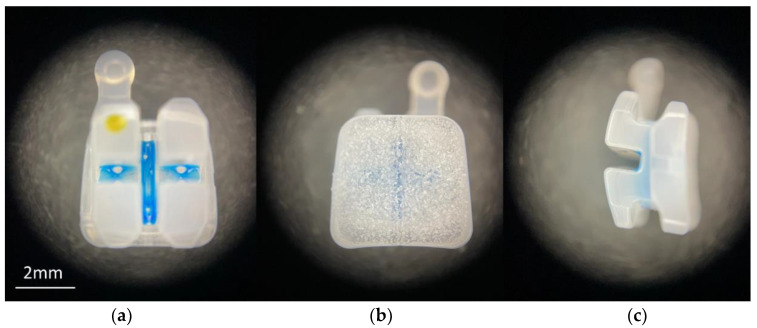
Ceramic bracket with microcrystalline base: (**a**) frontal view; (**b**) bracket base; and (**c**) lateral view.

Ceramic and metal brackets (Table 3 and Figure 1 and Figure 2) were positioned on enamel’s surface in accordance with MBT prescription [1] and pressed firmly into secured position with the uniform force (300 g) measured with Dontrix dynamometer (Acmedent, Concord, ON, Canada). After removal of the material excess around bracket base and bracket wings, the adhesive was polymerized for 3 s with the Curing Pen curing lamp (Eighteeth, Changzhou, China), with 5 W high-power blue-light-emitting diode (LED) with a wavelength of 380–515 nm.

### 2.2. Shear Bond Strength

**Figure 3:**
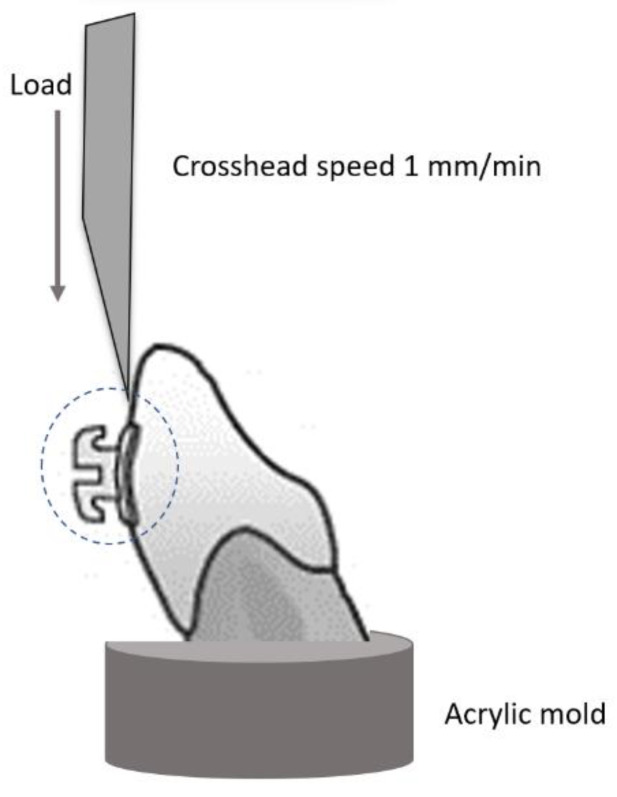
SBS test for orthodontic bracket.

Next, all specimens were stored in distilled water for 24 h at room temperature and thermocycled (5000 thermocycles; water baths of 5 °C and 55 °C; dwell time of 60 s). SBS was measured with the Zwick/Roell Z020 universal testing machine (Zwick-Roell, Ulm, Germany) at a crosshead speed of 1 mm/min (Figure 3). The load was parallel to the bracket base, and the shear force causing the fracture at enamel–bracket interface was registered. SBS expressed in megapascals (MPa) was calculated as follows: SBS [MPa] = shear force [N]/bracket base area [mm^2^].

### 2.3. ARI Score

After deboning, specimens were observed (mag. 20×) under BX51 optical microscope (Olympus, Tokyo, Japan) in search of potential damage on enamel’s surface. Moreover, the amount of residual adhesive was assessed according to Årtun’s ARI score [24,25] with criteria presented in Table 4.

### 2.4. Statistical Analysis

Obtained data were analysed using a one-way analysis of variation (ANOVA) to determine statistical significance of SBS values. Statistical significance was defined as *p* < 0.05 and calculated using a minimal sample size of 10. The power of the test was 0.98.

The Tukey’s post hoc test was used to determine which particular differences between groups of means were significant. To confirm the statistical significance of collected ARI score, non-parametric Kruskal–Wallis test was used. To assess the differences between the mean values of ARI in particular groups, post-hoc analysis with Mann–Whitney U test was performed.

Institutional Ethical Committee approval was obtained for this study (RNN/147/19/KE).

## 3. Results

### 3.1. Shear Bond Strength Measurements

**Figure 4:**
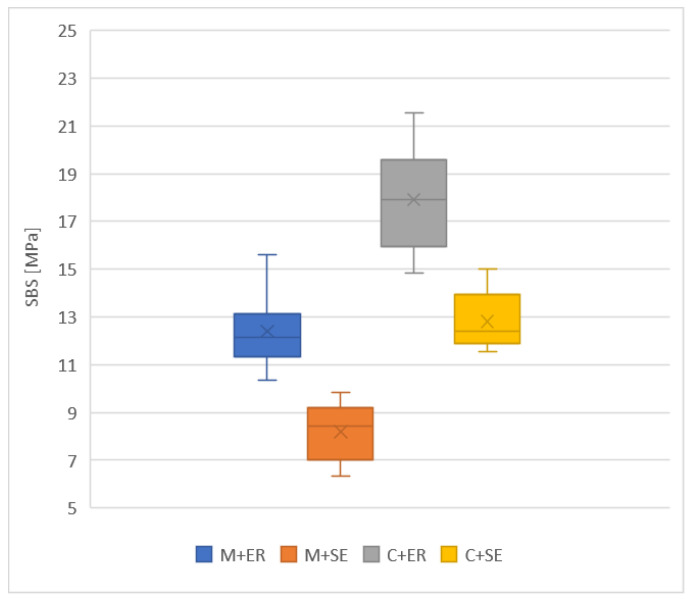
Mean SBS values (MPa).

The mean values of the SBS are presented in Figure 4 and Table 5.

C + ER exhibited the highest SBS values (17.93 ± 2.15 MPa), while the M + SE procedure achieved the lowest ones (8.19 ± 1.18 MPa). The mean values of SBS for C + ER were significantly higher than for the following groups: M + ER, M + SE and C + SE. Additionally, the mean values of SBS for M + SE were significantly lower than those for C + SE, C + ER and M + ER (*p* < 0.00001). The results of Tukey’s post hoc test are presented in Table 6.

### 3.2. ARI Score

**Figure 5:**
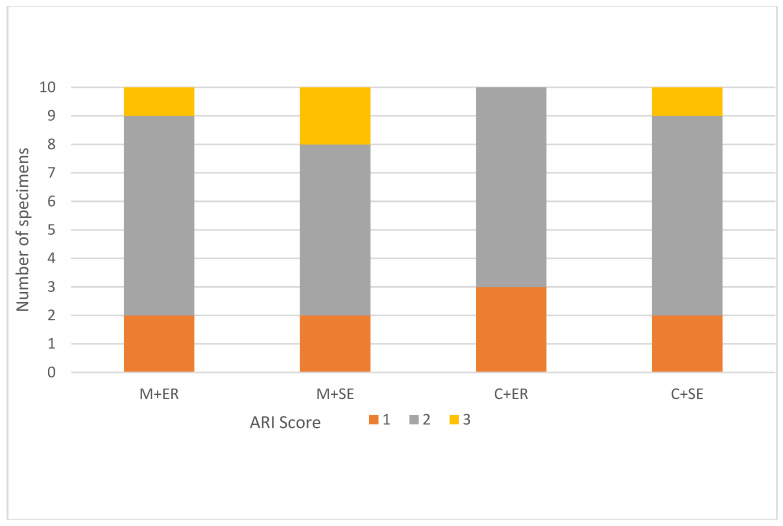
ARI scores.

**Figure 6:**
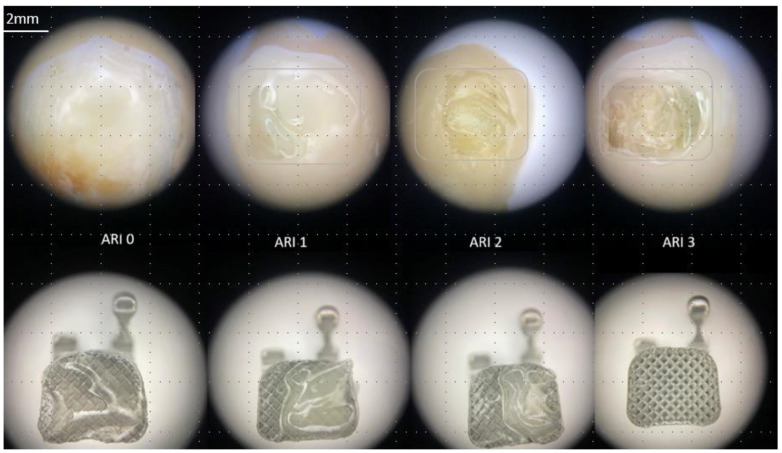
The representative images of ARI score (20× magnification).

Our post-debonding microscopic assessment (with a magnification of 20×) did not reveal the presence of enamel damage. The results of the ARI scores are presented in Figure 5. The Kruskal–Wallis test indicated that there was no significant difference in the ARI score between the study groups (*p* = 0.71). The most common ARI score was 2. In 21 specimens, more than 50% of the adhesive (ARI 2 or 3) remained on the enamel’s surface. ARI = 0 was not observed in all specimens. The representative microscopic image for each ARI value is presented in Figure 6.

## 4. Discussion

Bracket selection and bonding are the most important elements in orthodontic treatment. The clinician has to reconcile two aspects: the mechanical properties of orthodontic brackets and patient demand for short and efficient treatments without any discomfort in which the bracket appearance is acceptable. The properties of the most common orthodontic backets are presented in Table 7.

The aim of this study was to compare SBS metal and ceramic brackets bonded directly to the enamel’s surface, depending on different etching methods. The null hypothesis was rejected, as the results showed significant differences in the SBS between the ER and SE protocols, as well as between metal and ceramic brackets. It is worth emphasizing that significantly higher values of SBS for ceramic brackets in comparison to those for metal ones in both etching protocols were observed. However, the literature’s findings are ambiguous regarding this issue [6,26,27,28,29,30,31,32,33]. The majority of studies have confirmed the advantage of ceramic brackets over metal ones in terms of the SBS value [6,26,27,28,29,30] (Table 8).

Due to the increased base area of Clarity Advanced brackets (fine-grained quartz immersed in polycrystalline alumina), higher values of SBS were reached [6]. Hence, one research study assessing metal Victory Series and ceramic Clarity Advanced brackets using the ER protocol found lower values of the SBS of both metal and ceramic brackets versus the SBS achieved in the present study (Victory Series metal brackets—5.63 MPa vs. 12.40 MPa; Clarity Advanced ceramic brackets—8.98 MPa vs. 17.92 MPa, respectively) [26]. These lower values could be associated with different methods of specimen storage and material aging simulations. In the present study, before thermocycling, the specimens were stored in distilled water for 24 h, whereas Elsaka et al. [26] stored them for longer (1 week) in different conditions (artificial saliva). Although the present study used a longer thermocycling protocol in comparison to the abovementioned one (5000 cycles and a dwell time of 60 s vs. 1000 cycles and a dwell time of 30 s), higher SBS values were achieved. It is worth mentioning that each group of 500 cycles reduces the SBS values by about 16.7% [31,34]. According to the literature, specimens should be cycled a minimum of 5000 times with a 60 s dwell time [32,33]; therefore, this protocol was used in the present study. However, most researchers apply their own regimen of thermocycling [26,27,28,29,30], because the values included in ISO TR 11450 (500 cycles, 5 and 55 °C) are considered to be insufficient to achieve the proper aging effect [32,33].

Yadav et al. [27] and Rahul et al. [28] used metal and ceramic brackets with Transbond XT with the ER protocol as a control groups for testing the effect of enamel bleaching on the SBS. Apart from the decrease in the SBS after bleaching, a significant difference in the SBS between ceramic (13.80 MPa) and metal brackets in the control group (12.18 MPa) was found. Bilen et al. [29] found a higher SBS of Clarity Advanced brackets regardless of the etching method (SE or ER). A particularly important finding of the above-mentioned study was the higher microleakage in the metal bracket which could result in a lower SBS due to an incomplete polymerization of the orthodontic adhesive. The metallic structure, in contrast to the translucent ceramic bracket, limits the light penetration from the curing lamp. It is worth mentioning that all the SBS values noted by Bilen et al. were noticeably higher than the values found in the present study (18.47 MPa vs. 12.40 MPa for M-ER and 21.47 vs. 17.92 MPa and for C-ER, respectively) [29]. This difference may result from the lack of a thermocycling procedure in the above-mentioned study. Another reason causing this discrepancy in results could be that metal brackets with larger base areas (Gemini metal brackets—10.61 mm^2^ vs. Victory Series metal brackets—8.97 mm^2^) were tested [29].

Metal and ceramic brackets from AO (American Orthodontics, Sheboygan, USA), with bracket base areas of 11.3 mm^2^ and 15.1 mm^2^, respectively, were also investigated [30,31]. Similarly to the present study, the ER protocol and Transbond XT were used. One of the above-mentioned studies revealed no statistical difference in the SBS between ceramic (14.62 MPa) and metal brackets (13.71 MPa) [30]. However, the other one proved that metal brackets exhibited a significantly higher SBS (24.92 MPa) in comparison to that of ceramic ones (10.74 MPa) [31]. The inferiority of ceramic brackets is not supported by the present study and could result from the lack of micromechanical retention on the base of AO brackets [31].

The present study also revealed the difference in the SBS depending on the etching protocol. Ceramic brackets bonded with ER achieved a significantly higher SBS (17.92 MPa) than that using the SE protocol (12.81 MPa). The same phenomenon was observed for metal brackets (12.4 MPa vs. 8.4 MPa), because ER provides optimal surface development for the penetration of orthodontic adhesive. However, its main disadvantages are its number of clinical steps and prolonged chair-side time. It can result in a higher risk of operator’s error, such as the moisture contamination of the etched surface, leading to the bracket’s failure. On the other hand, the SE technique simplified the bonding process as the etchant and primer are applied in one step without rinsing. However, the SE acid monomers do not penetrate the enamel as deeply as the adhesive resin applied after the ER protocol. The superficial incorporation of the enamel results in lower SBS values [35].

Moreover, the risk of enamel damage is a main concern and depends on the location of the adhesive fracture. In the majority of the tested specimens, the bond failure occurring at the bracket–adhesive interface (an ARI score of 2) was more favourable to maintain the enamel’s undestroyed surface. Our optical microscopic evaluation (a mag of 20×) did not reveal any cracks or damages on the enamel’s surface that could result from a cohesive fracture (in all study groups). Moreover, the etching protocol did not influence the risk of enamel failure, although the SE technique resulted in a weaker adhesion. However, the adhesive material remaining on the tooth’s surface indicates a prolonged removal time and surface polishing [2,20]. The ARI scores presented in the literature endorse the notion that the bond strength values are correlated with the amount of material left on the enamel’s surface after debonding. Higher SBS values of ceramic brackets corresponded, in the majority of research, with an ARI 2 or 3 [6,26,27,28,29,30]. In present research, the ARI values were not correlated with the SBS.

Contemporary metal and ceramic brackets became widespread in orthodontics along with the development of etching and bonding techniques [18,25,36,37]. The choice of brackets (Victory Series and Clarity Advanced) for this study was determined by the bracket base area and its structure. Victory Series metal brackets have a relatively small base area (8.97 mm^2^) with a foil mesh retentive surface. Moreover, their austenitic stainless-steel structure is the most commonly investigated one [1,37]. On the other hand, Clarity Advanced ceramic brackets, despite their larger bracket base area (11.69 mm^2^), are preferred by patients with high aesthetic demands, because the appliance is transparent and the attachments are less visible [1,6,8,16,38]. Additionally, the choice of adhesive (Transbond XT) was based on the most frequent material used in the literature and applied in clinical practice [26,29,31,36,37,39,40,41,42]. Thus, this light-cured adhesive combined with the ER technique serves as a point of reference for in vitro tests [6,26,29,31,36,37,39,40,41,42,43].

An SBS of 5.9–7.8 MPa has become the benchmark for adhesion in orthodontics since its introduction in 1975 by Reynolds [37,39,40,43]. This value is of high clinical importance because the probability of dental tissue impairment increases 1.3 times per one additional MPa of SBS [35]. The values presented by Reynolds are almost two times lower in comparison to those of the present study and to the results obtained by other researchers in the last decade. However, if the one-digit SBS value was sufficient in 70 s, it will also remain satisfactory using current, improved dental materials and bonding techniques [26,31,36,37,39,40,41,42,43]. Therefore, the bracket–enamel bond strength should be wisely adjusted according to clinical indications. The decreased bond strength can be applied in cases with enamel defects such as hypoplasia, hypomineralization, cracks or restorations. This is also advisable when the treatment plan includes bracket repositioning during treatment, for instance, in patients with severe dental crowding, improper shape of dental crowns or extended attrition. On the other hand, a higher bond strength should be applied when the bracket is exposed to an increased load or attached to orthodontic springs, loops or elastics. Recurring bracket failure can also be an indication for changing the bracket type [1,6,8,16,38].

The limitations of the present study are associated with in vitro conditions that do not ideally reflect the intraoral situation. Although there is no evidence that the non-vital post-extraction tissue condition can affect the values of SBS, other possible variables were considered, such as thermocycling and the occlusal load [44,45,46,47,48,49,50,51,52,53]. Even though there are methods to simulate the aging of adhesive (thermocycling), it is impossible to reflect the natural bracket–enamel interaction, in which the bond is exposed to saliva, oral biofilm and mechanical load (orthodontic wire and occlusion) [44,45,46,47,48,49,50,51,52,53]. Moreover, the load during intraoral debonding is not homogenous and can even be five times greater than the linear load created with a universal testing machine [44]. For that reason, the main drawback of the present study is the limited possibility of extrapolating and confronting the obtained SBS values with results achieved in clinical research. To supplement the present study, a thorough qualitative and quantitative analysis of the enamel’s surface after debonding could be performed using microscopic techniques, such as confocal laser scanning microscopy (CLSM), transmission electron microscopy (TEM), scanning electron microscopy (SEM) and atomic force microscopy (AFM), to assess the post-debonding enamel microtopography [25,37]. Moreover, spectroscopic techniques such as energy-dispersive spectroscopy (SEM-EDX), Fourier-transform infrared spectroscopy (FTIR) or Raman’s spectroscopy could be implemented to obtain a greater magnification and resolution and to analyse changes in the elemental composition of the enamel and bracket surface after debonding [25,40]. It is worth mentioning that alternative methods of enamel conditioning (sandblasting, an Er:YAG laser or etchants containing β-tricalcium phosphate and monocalcium phosphate monohydrate powders) and treatment methods for the bracket’s surface (adhesive precoated brackets vs. operator-coated brackets) should also be evaluated [45,46]. Additionally, self-ligating brackets or the lingual technique demands a thorough evaluation using different etching and bonding protocols. As clinical situations often demand bonding brackets to different dental materials’ adhesion to composite, ceramic or metal restorations also should be tested [54,55,56]. An assessment of the clinical performance is required to provide well-grounded recommendations for orthodontists.

**Table 8:** SBS values of metal and ceramic brackets bonded with etch-and-rinse protocol and Transbond XT adhesive.

Author, Year	Type of the Brackets	Bracket Base Area [mm^2^]	Number of Specimens	SBS Results [MPa]
Bilen et al., 2020 [29]	Metal: Gemini, 3M Unitek, Monrovia, CA, USA	10.61	12	18.47 ± 4.46
	Ceramic (Clarity Advanced, 3M Unitek, Monrovia, CA, USA)	11.69	12	21.47 ± 7.75
Delavarian et al., 2019 [30]	Metal: American Orthodontics, Sheboygan, WI, USA	11.3	30	13.71 ± 3.54
	Ceramic (American Orthodontics, WI, USA)	15.1	30	14.62 ± 4.30
Almoammar et al., 2019 [50]	Metal (Ovation, GAC, Dentsply Sirona, NC, USA)	10.89	10	14.84 ± 1.78
	Ceramic (Allure III, Dentsply Sirona, NC, USA)	11.89	10	12.52 ± 1.28
Yadav et al., 2018 [27]	Metal Ormco Mini 2000 series, Ormco Corporation, Glendora, CA, USA	8.79	10	12.18 + 1.41
	Ceramic Ormco Inspire Ice series, Ormco Corporation, Glendora, CA	11.7	10	13.80 + 1.69
Rahul et al., 2017 [28]	Metal (no data)	NA	12	16.03 + 0.87
	Ceramic (no data)	NA	12	20.21 + 0.94
Ansari et al., 2018 [6]	Metal Gemini Unitek/3M, Monrovia, CA, USA	10.30	10	17.50 ± 2.41
	Ceramic Clarity Advanced Unitek/3M, Monrovia, California	11.69	10	27.26 ± 1.73
Chalipa et al., 2016 [31]	Metal (American Orthodontics, WI, USA)	11.3	10	24.92 ± 6.37
	Ceramic (American Orthodontics, WI, USA)	15.1	10	10.74 ± 3.18
Pinho et al., 2020 [41]	Metal	NA	10	6.9 ± 3.2
	Ceramic	NA	10	4.7 ± 1.5
Elsaka et al., 2015 [26]	Metal (Victory Series, 3M)	8.97	10	5.63 + 0.79
	Ceramic (Clarity 3M)	11.69	10	8.98 + 1.5

NA: not available.

## 5. Conclusions

Within the limitations of the present study, the following conclusions can be drawn:

1. Ceramic brackets achieved the highest SBS values in both the SE and ER protocols.
2. Higher SBS values for ceramic and metallic brackets were found in the ER protocol.
3. In all tested groups, the achieved SBS value was satisfactory to withstand orthodontic and occlusal forces.
4. There was no significant difference in the ARI score between study groups (*p* = 0.71). The interface between the bracket base and adhesive was the focus of minor resistance which probably decreased the risk of enamel damage during debonding.

Clinical significance: Ceramic brackets cemented with the etch-and-rinse protocol are recommended in cases in which higher forces are required, for example, for patients with increased occlusal load due to severe crowding. In case of enamel defects, metallic brackets and the SE protocol should be chosen.

## Figures and Tables

**Table 1:** Study groups.

	Etch-and-Rinse Technique		Self-Etch Technique	
	M + ER	C + ER	M + SE	C + SE
Enamel l	H_3_PO_4_		Transbond Plus Self-etching Primer	
	Rinsing and drying		Gentle drying	
Enamel + bracket base	Transbond XT Primer		NA	
Bracket base	Transbond XT Light Cure Adhesive			
Enamel+ bracket base	Bracket placement			
	Light-curing			

M + ER—metal brackets + etch-and-rinse; C + ER—ceramic brackets + etch-and-rinse; M + SE—metal brackets + self-etch; C + SE—ceramic brackets + self-etch; NA—not applicable.

**Table 2:** Materials used in the study.

Material	Composition	Application Protocol	Time of Application
Scotchbond Universal Etchant (3M ESPE, St. Paul, MN, USA)	32% wg. H_3_PO_4_	A uniform layer on cleaned and dried enamel for 30 s.	30 s followed with 30 s of rinsing and 15 s of drying
Transbond XT Primer (3M Unitek, Monrovia, CA, USA)	TEGDMA, Bis-GMA	A thin coat on a chalky white etched enamel surface	NA
Transbond Plus Self Etching Primer (3M, Unitek, Monrovia, CA, USA)	MPP, EGDMA, H_3_PO_4_, HEMA, acrylic acid, MED-ester, mono HEMA, tBMEP, CQ	Rubbing (with the applicator tip) the cleaned and dried enamel.	3–5 s per tooth followed with a gentle air burst for 1–2 s
Transbond XT (3M Unitek, Monrovia, CA, USA)	silane-treated quartz, Bis-GMA, dichlorodimethylsilane reaction product with silica; 77% quartz (silica) filler	A uniform layer applied on bracket base	NA

H_3_PO_4_—phosphoric acid; TEGDMA—Triethylene Glycol Dimethacrylate; Bis-GMA—Bisphenol A Diglycidyl Ether Dimethacrylate; MPP—Methacrylated pyrophosphates; EGDMA—Ethylene Glycol Dimethacrylate, HEMA-, 2-hydroxyethyl methacrylate; MED-ester—Methyl-phosphinicobis, (oxy-2,1-ethandiyl)ester; tBMEP—tris [2-(methacryloyloxy)ethyl]phosphate; CQ—camphorquinone; NA—not applicable.

**Table 3:** Brackets used in this study.

Manufacturer	Bracket Type	Internal Structure	Prescription	Base Type	Base Area
Victory Series™ (3M, Unitek, Monrovia, CA, USA)	Metal	Austenitic stainless steel	0.022” MBT for upper premolar	Foil mesh	8.97 mm^2^
Clarity™ Advanced (3M, Unitek, Monrovia, CA, USA	Ceramic	Polycrystalline	0.022” MBT for upper premolar	Microcrystalline mechanical	11.69 mm^2^

**Table 4:** Årtun’s ARI Index.

ARI Score	Interpretation	Enamel Surface
0	No adhesive left on the tooth	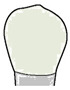
1	Less than half of the adhesive left on the tooth	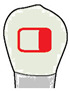
2	More than half of the adhesive left on the tooth	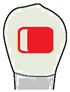
3	All the adhesive left on the tooth, with a distinct impression of the bracket mesh	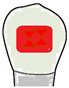

**Table 5:** Results of one-way ANOVA for study groups.

	Mean SBS	Median	Min	Max	SD	F-Value	*p*-Value
M + ER	12.40	12.15	10.33	15.60	1.56	63.97	*p* < 0.00001
M + SE	8.19	8.44	6.35	9.82	1.18		
C + ER	17.93	17.93	14.85	21.56	2.15		
C + SE	12.82	12.39	11.54	15.03	1.22		

Mean SBS—mean shear bond strength; Min—minimum SBS value; Max—maximum SBS value; SD—standard deviation. Statistical significance was defined as *p* < 0.05.

**Table 6:** Results of Tukey’s test.

Group	Difference	Q	*p*-Value
M + ER vs. M + SE	4.217	8.4568	0.00000
M + ER vs. C + ER	5.523	11.0759	0.00000
M + ER vs. C + SE	0.412	0.8262	0.9362
M + SE vs. C + ER	9.74	19.5327	0.00000
M + SE vs. C + SE	4.629	9.283	0.00000
C + ER vs. C + SE	5.111	10.2496	0.00000

**Table 7:** Comparison of metal, polycrystalline and monocrystalline brackets [1,6,9,10,11,12,13,14,15].

Feature	Metal Stainless-Steel Bracket	Polycrystalline Bracket	Monocrystalline Bracket
Aesthetics	Silver colour	Translucent	Transparent
Internal structure	Mostly austenitic crystalline structure	20–30 µm particles of Al_2_O_3_ (inhomogeneous)	A single large crystal rod of Al_2_O_3_
Tensile strength	210 MPa	380 MPa	1800 MPa
Manufacturing	Metal injection moulding (MIM) or computer numerated controlled (CNC) milling	Ceramic injection moulding (CIM) or sintering blended particles of Al_2_O_3_ at a temp. of 1800 °C, followed by cooling and drill cutting to provide bracket shape	Melting Al_2_O_3_ above 2100° and slowly cooling to control crystallization; machine or laser cutting to provide the bracket shape
Fracture characteristics	Base and wings remain integrated during debonding	Limited crack propagation Less prone to fracture	Easier crack propagation High risk of bracket fracture during debonding
Debonding recommendation	Mechanical with squeezing and peel-off force applied with pilers to the base or wings	Mechanical—possible only with recommended pilers Ultrasonic, chemical or laser methods—more preferable	
Fracture resistance after aging	Weakens due to corrosion	Remains unchanged	Weakens
Coefficient of friction with 0.019 × 0.025” SS wire	0.21	0.48	0.42

## Data Availability

The data presented in this study are available on request from the corresponding author.
